# Supplementary material for: Problematic Gaming during COVID-19 Pandemic: A Systematic Review, Meta-Analysis, and Meta-Regression
Source: Healthcare (Basel). 2023 Dec 15;11(24):3176. doi: 10.3390/healthcare11243176 (PMC10742917; doi:10.3390/healthcare11243176)
Supplement: Supplementary file 1 [file healthcare-11-03176-s001.zip › S2 - Search strategy.pdf]

Supplementary S2. Search strategy.

GOOGLE SCHOLAR: string search

(COVID-19 OR coronavirus OR 2019-ncov OR sars-cov-2 OR lockdown) AND (gaming OR videogam*)

MEDLINE (EBSCO): string search (example under Abstracts)

AB covid-19 AND AB coronavirus AND AB 2019-ncov AND AB sars-cov-2 AND AB lockdown OR AB gaming AND AB videogam*

PUBMED: string search

((((((covid-19[Title/Abstract]) OR (coronavirus[Title/Abstract])) OR (2019-ncov[Title/Abstract])) OR (sars-cov-2[Title/Abstract])) OR (lockdown[Title/Abstract])) AND (gaming[Title/Abstract])) OR (videogam*[Title/Abstract])

SCIENCE DIRECT: string search

(COVID-19 OR coronavirus OR 2019-ncov OR sars-cov-2 OR lockdown) AND (gaming OR videogame)

SCOPUS: string search

(COVID-19 OR coronavirus OR 2019-ncov OR sars-cov-2 OR lockdown) AND (gaming OR videogam*)
