# Supplementary material for: Problematic Gaming during COVID-19 Pandemic: A Systematic Review, Meta-Analysis, and Meta-Regression
Source: Healthcare (Basel). 2023 Dec 15;11(24):3176. doi: 10.3390/healthcare11243176 (PMC10742917; doi:10.3390/healthcare11243176)
Supplement: Supplementary file 1 [file healthcare-11-03176-s001.zip › S3 - Contacted authors.pdf]

Supplementary S3. List of contacted authors.

First database search (23 June, 2022)

Reference	Date	Data retrieved
**Balhara et al., 2020 (198)**	7-09-22	Sample characteristics
**Brailovskaia et al., 2022 (229)**	17-08-22	Sample characteristics
**Chen et al., 2021 (143)**	10-08-22	Sample characteristics
**Chen et al., 2021 (156)**	10-08-22	Sample characteristics
**Chen et al., 2021 (212)**	10-08-22; 7-09-22	Sample characteristics
**Ciccarelli et al., 2022 (219)**	17-08-22	Sample characteristics
**Ismail et al., 2021 (207)**	10-08-22	Sample characteristics
**Rozgonjuk et al., 2022 (194)**	17-08-22; 11-10-22	None
**Sallie et al., 2021 (214)**	7-09-22	Sample characteristics
**Volpe et al., 2022 (199)**	17-08-22; 14-10-22	Sample characteristics
**Wu et al., 2022 (208)**	17-08-22; 07-07-22	None

Second database search (5 December, 2022)

Reference	Date	Data retrieved
**Bersani et al., 2022 (253)**	05-12-22	None
**Chen et al., 2022 (3) (255)**	05-12-22	Sample characteristics
**Jouhki et al., 2022 (256)**	05-12-22	None
**Sousa Tavares et al., 2022 (257)**	05-12-22	None
**Xiang et al., 2022 (258)**	05-12-22	None
**Yang et al., 2022 (259)**	05-12-22	None

References

Balhara, Y. S., Kattula, D., Singh, S., Chukkali, S., & Bhargava, R. (2020). Impact of lockdown following COVID-19 on the gaming behavior of college students. *Indian Journal of Public Health*, *64*(6), 172. https://doi.org/10.4103/ijph.IJPH_465_20

Bersani, F. S., Barchielli, B., Ferracuti, S., Panno, A., Carbone, G. A., Massullo, C., Farina, B., Corazza, O., Prevete, E., Tarsitani, L., Pasquini, M., Biondi, M., & Imperatori, C. (2022). The association of problematic use of social media and online videogames with aggression is mediated by insomnia severity: A cross-sectional study in a sample of 18- to 24-year-old individuals. *Aggressive Behavior*, *48*(3), 348–355. https://doi.org/10.1002/ab.22008

Brailovskaia, J., Meier-Faust, J., Schillack, H., & Margraf, J. (2022). A two-week gaming abstinence reduces Internet Gaming Disorder and improves mental health: An experimental longitudinal intervention study. *Computers in Human Behavior*, *134*, 107334. https://doi.org/10.1016/j.chb.2022.107334

Chen, C.-Y., Chen, I.-H., O'Brien, K. S., Latner, J. D., & Lin, C.-Y. (2021). Psychological distress and internet-related behaviors between schoolchildren with and without overweight during the COVID-19 outbreak. *International Journal of Obesity*, *45*(3), 677–686. https://doi.org/10.1038/s41366-021-00741-5

Chen, C.-Y., Chen, I.-H., Pakpour, A. H., Lin, C.-Y., & Griffiths, M. D. (2021). Internet-related behaviors and psychological distress among schoolchildren during the COVID-19 school hiatus. *Cyberpsychology, Behavior, and Social Networking*, *24*(10), 654–663. https://doi.org/10.1089/cyber.2020.0497

Chen, I. H., Chen, C. Y., Liu, C. H., Ahorsu, D. K., Griffiths, M. D., Chen, Y. P., Kuo, Y. J., Lin, C. Y., Pakpour, A. H., & Wang, S. M. (2021). Internet addiction and psychological distress among Chinese schoolchildren before and during the COVID-19 outbreak: A latent class analysis. *Journal of Behavioral Addictions*, *10*(3), 731–746. https://doi.org/10.1556/2006.2021.00052

Chen, I., Chen, H., Gamble, J., Liao, X., Chen, X., Yang, Y., Pakpour, A., Griffiths, M., & Lin, C. (2022). Evaluating a cross-lagged panel model between problematic internet use and psychological distress and cross-level mediation of school administrator support on problematic internet use: The serial mediating role of psychological needs thwarting of online teaching and psychological distress. *Frontiers in Public Health*, *10*, 987366. https://doi.org/10.3389/fpubh.2022.987366

Ciccarelli, M., Nigro, G., D'Olimpio, F., Griffiths, M. D., Sacco, M., Pizzini, B., & Cosenza, M. (2022). The associations between loneliness, anxiety, and problematic gaming behavior during the COVID-19 pandemic: The mediating role of mentalization. *Mediterranean Journal of Clinical Psychology*, *Vol 10*, No 1 (2022). https://doi.org/10.13129/2282-1619/MJCP-3257

Ismail, N., Tajjudin, A. I., Jaafar, H., Nik Jaafar, N. R., Baharudin, A., & Ibrahim, N. (2021). The relationship between Internet addiction, Internet gaming and anxiety among medical students in

a Malaysian public university during COVID-19 pandemic. *International Journal of Environmental Research and Public Health*, *18*(22), 11870. https://doi.org/10.3390/ijerph182211870

Jouhki, H., Savolainen, I., Sirola, A., & Oksanen, A. (2022). Escapism and excessive online behaviors: A three-wave longitudinal study in Finland during the COVID-19 pandemic. *Internation Journal of Environmental Research and Public Health*, *19*(19). https://doi.org/10.3390/ijerph191912491

Rozgonjuk, D., Pontes, H. M., Schivinski, B., & Montag, C. (2022). Disordered gaming, loneliness, and family harmony in gamers before and during the COVID-19 pandemic. *Addictive Behaviors Reports*, *15*, 100426. https://doi.org/10.1016/j.abrep.2022.100426

Sallie, S. N., Ritou, V. J. E., Bowden-Jones, H., & Voon, V. (2021). Assessing online gaming and pornography consumption patterns during COVID-19 isolation using an online survey: Highlighting distinct avenues of problematic internet behavior. *Addictive Behaviors*, *123*, 107044. https://doi.org/10.1016/j.addbeh.2021.107044

Sousa Tavares, H. L., Souza Costa, D., Soares, A. A., Kestelman, I., da Silva, A. G., Malloy-Diniz, L. F., de Paula, J. J., & de Miranda, D. M. (2022). Gaming addiction and screen time in a context of increase of internalizing symptoms: Moderation evaluation. *Clinical Child Psychology and Psychiatry*, 13591045221125328. https://doi.org/10.1177/13591045221125329

Volpe, U., Orsolini, L., Salvi, V., Albert, U., Carmassi, C., Carrà, G., Cirulli, F., Dell'Osso, B., Luciano, M., Menculini, G., Nanni, M., Pompili, M., Sani, G., Sampogna, G., Group, W., & Fiorillo, A. (2022). COVID-19-related social isolation predispose to problematic Internet and online video gaming use in Italy. *International Journal of Environmental Research and Public Health*, *19*(3), 1539. https://doi.org/10.3390/ijerph19031539

Wu, Q., Luo, T., Tang, J., Wang, Y., Wu, Z., Liu, Y., Chen, W., Deng, Q., & Liao, Y. (2022). Gaming in China before the COVID-19 pandemic and after the lifting of lockdowns: A nationwide online retrospective survey. *International Journal of Mental Health and Addiction*. https://doi.org/10.1007/s11469-022-00792-3

Xiang, G., Gan, X., Jin, X., & Zhang, Y. (2022). The more developmental assets, the less internet gaming disorder? Testing the cumulative effect and longitudinal mechanism during the COVID-19 pandemic. *Current Psychology*, 1–12. https://doi.org/10.1007/s12144-022-03790-9

Yan, E., Sun, R., Wu, A., Lai, D., & Lee, V. (2022). The impact of pandemic-related life stress on Internet gaming: Social cynicism and gaming motivation as serial mediators. *International Journal of Environmental Research and Public Health*, *19*(14). https://doi.org/10.3390/ijerph19148332
