# Supplementary material for: Problematic Gaming during COVID-19 Pandemic: A Systematic Review, Meta-Analysis, and Meta-Regression
Source: Healthcare (Basel). 2023 Dec 15;11(24):3176. doi: 10.3390/healthcare11243176 (PMC10742917; doi:10.3390/healthcare11243176)
Supplement: Supplementary file 1 [file healthcare-11-03176-s001.zip › S4 - Included.pdf]

Supplementary S4. List of included references (*N* = 62).

1. Alqassim, A., S. Mahfouz, M., M. Hakami, M., A. Al Faqih, A., A. Shugairi, A., R. Alsanosy, M., Y. Rayyani, A., Y. Albrraq, A., A. Muaddi, M., & A. Alharbi, A. (2022). Depression, anxiety, stress and their association with the use of electronic devices among adolescents during the COVID-19 pandemic. *International Journal of Mental Health Promotion*, *24*(2), 251–262. https://doi.org/10.32604/ijmhp.2022.019000
2. Al-Sharqi, M. A., & Hasan, H. S. (2022). Neural network to investigate gaming addiction and its impact on health effects during the COVID-19 Pandemic. *Periodicals of Engineering and Natural Sciences (PEN)*, *10*(1), 504. https://doi.org/10.21533/pen.v10i1.2713
3. Balhara, Y. S., Kattula, D., Singh, S., Chukkali, S., & Bhargava, R. (2020). Impact of lockdown following COVID-19 on the gaming behavior of college students. *Indian Journal of Public Health*, *64*(6), 172. https://doi.org/10.4103/ijph.IJPH_465_20
4. Brailovskaia, J., Meier-Faust, J., Schillack, H., & Margraf, J. (2022). A two-week gaming abstinence reduces Internet Gaming Disorder and improves mental health: An experimental longitudinal intervention study. *Computers in Human Behavior*, *134*, 107334. https://doi.org/10.1016/j.chb.2022.107334
5. Cakiroglu, S., Soylu, N., Department of Child and Adolescent Psychiatry, Istanbul University Istanbul School of Medicine, Istanbul, Turkey, Gormez, V., & Department of Child and Adolescent Psychiatry, Istanbul Medeniyet University Goztepe Training and Research Hospital, Istanbul, Turkey. (2021). Re-evaluating the digital gaming profiles of children and adolescents during the COVID-19 pandemic: A comparative analysis

comprising 2 years of pre-pandemic data. *Addicta: The Turkish Journal on Addictions*, *8*(1), 51–57. https://doi.org/10.5152/ADDICTA.2021.21019

6. (Update) Cena, L., Rota, M., Calza, S., Trainini, A., Zecca, S., Zappa, S. B., Nodari, L. S., & Stefana, A. (2022). Prevalence and types of video gaming and gambling activities among adolescent public school students: Findings from a cross-sectional study in Italy. *Italian Journal of Pediatrics*, *48*(1), 108. https://doi.org/10.1186/s13052-022-01299-2

7. Chang, C. I., Fong Sit, H., Chao, T., Chen, C., Shen, J., Cao, B., Montag, C., Elhai, J. D., & Hall, B. J. (2022). Exploring subtypes and correlates of internet gaming disorder severity among adolescents during COVID-19 in China: A latent class analysis. *Current Psychology*. https://doi.org/10.1007/s12144-022-03133-8

8. Chen, C.-Y., Chen, I.-H., Hou, W.-L., Potenza, M. N., O'Brien, K. S., Lin, C.-Y., & Latner, J. D. (2022). The relationship between children's problematic Internet-related behaviors and psychological distress during the onset of the COVID-19 pandemic: A longitudinal study. *Journal of Addiction Medicine*, *16*(2), e73–e80. https://doi.org/10.1097/ADM.0000000000000845

9. Chen, C.-Y., Chen, I.-H., O'Brien, K. S., Latner, J. D., & Lin, C.-Y. (2021). Psychological distress and internet-related behaviors between schoolchildren with and without overweight during the COVID-19 outbreak. *International Journal of Obesity*, *45*(3), 677–686. https://doi.org/10.1038/s41366-021-00741-5

10. Chen, C.-Y., Chen, I.-H., Pakpour, A. H., Lin, C.-Y., & Griffiths, M. D. (2021). Internet-related behaviors and psychological distress among schoolchildren during the COVID-19 school hiatus. *Cyberpsychology, Behavior, and Social Networking*, *24*(10), 654–663. https://doi.org/10.1089/cyber.2020.0497

11. Chen, I. H., Chen, C. Y., Liu, C. H., Ahorsu, D. K., Griffiths, M. D., Chen, Y. P., Kuo, Y. J., Lin, C. Y., Pakpour, A. H., & Wang, S. M. (2021). Internet addiction and psychological distress among Chinese schoolchildren before and during the COVID-19 outbreak: A latent class analysis. *Journal of Behavioral Addictions*, *10*(3), 731–746. https://doi.org/10.1556/2006.2021.00052

12. (Update) Chen, I., Chen, H., Gamble, J., Liao, X., Chen, X., Yang, Y., Pakpour, A., Griffiths, M., & Lin, C. (2022). Evaluating a cross-lagged panel model between problematic internet use and psychological distress and cross-level mediation of school administrator support on problematic internet use: The serial mediating role of psychological needs thwarting of online teaching and psychological distress. *Frontiers in Public Health*, *10*, 987366. https://doi.org/10.3389/fpubh.2022.987366

13. Chen, I.-H., Chen, C.-Y., Pakpour, A. H., Griffiths, M. D., Lin, C.-Y., Li, X.-D., & Tsang, H. W. H. (2021). Problematic Internet-related behaviors mediate the associations between levels of internet engagement and distress among schoolchildren during COVID-19 lockdown: A longitudinal structural equation modeling study. *Journal of Behavioral Addictions*, *10*(1), 135–148. https://doi.org/10.1556/2006.2021.00006

14. Chen, I.-H., Lin, Y.-C., Lin, C.-Y., Wang, W.-C., & Gamble, J. H. (2022). The trajectory of psychological distress and problematic Internet gaming among primary school boys: A longitudinal study across different periods of COVID-19 in China. *Journal of Men's Health*, *18*(3), 070. https://doi.org/10.31083/j.jomh1803070

15. Ciccarelli, M., Nigro, G., D'Olimpio, F., Griffiths, M. D., Sacco, M., Pizzini, B., & Cosenza, M. (2022). The associations between loneliness, anxiety, and problematic gaming behavior during the COVID-19 pandemic: The mediating role of mentalization.

Mediterranean Journal of Clinical Psychology, *Vol 10*, No 1 (2022). https://doi.org/10.13129/2282-1619/MJCP-3257

16. Claesdotter-Knutsson, E., André, F., & Håkansson, A. (2022). Gaming activity and possible changes in gaming behavior among young people during the COVID-19 pandemic: Cross-sectional online survey study. *JMIR Serious Games*, *10*(1), e33059. https://doi.org/10.2196/33059
17. Cuong, V. M., Assanangkornchai, S., Wichaidit, W., Minh Hanh, V. T., & My Hanh, H. T. (2021). Associations between gaming disorder, parent-child relationship, parental supervision, and discipline styles: Findings from a school-based survey during the COVID-19 pandemic in Vietnam. *Journal of Behavioral Addictions*, *10*(3), 722–730. https://doi.org/10.1556/2006.2021.00064
18. De Pasquale, C., Chiappedi, M., Sciacca, F., Martinelli, V., & Hichy, Z. (2021). Online videogames use and anxiety in children during the COVID-19 pandemic. *Children*, *8*(3), 205. https://doi.org/10.3390/children8030205
19. Donati, M. A., Guido, C. A., De Meo, G., Spalice, A., Sanson, F., Beccari, C., & Primi, C. (2021). Gaming among children and adolescents during the COVID-19 lockdown: The role of parents in time spent on video games and gaming disorder symptoms. *International Journal of Environmental Research and Public Health*, *18*(12), 6642. https://doi.org/10.3390/ijerph18126642
20. Efrati, Y., & Spada, M. M. (2022). Self-perceived substance and behavioral addictions among Jewish Israeli adolescents during the COVID-19 pandemic. *Addictive Behaviors Reports*, *15*, 100431. https://doi.org/10.1016/j.abrep.2022.100431

21. Elhai, J. D., McKay, D., Yang, H., Minaya, C., Montag, C., & Asmundson, G. J. G. (2021). Health anxiety related to problematic smartphone use and gaming disorder severity during COVID-19: Fear of missing out as a mediator. *Human Behavior and Emerging Technologies*, *3*(1), 137–146. https://doi.org/10.1002/hbe2.227

22. Elsayed, W. (2021). Covid-19 pandemic and its impact on increasing the risks of children's addiction to electronic games from a social work perspective. *Heliyon*, *7*(12), e08503. https://doi.org/10.1016/j.heliyon.2021.e08503

23. Fernandes, B., Nanda Biswas, U., Tan-Mansukhani, R., Vallejo, A., & Essau, C. A. (2020). The impact of COVID-19 lockdown on Internet use and escapism in adolescents. *Revista de Psicología Clínica Con Niños y Adolescentes*, *7*(nº 3), 59–65. https://doi.org/10.21134/rpcna.2020.mon.2056

24. Giardina, A., Di Blasi, M. D. B., Schimmenti, A., King, D. L., Starcevic, V., & Billieux, J. (2021). Online gaming and prolonged self-isolation: Evidence from Italian gamers during the COVID-19 outbreak. *Clinical Neuropsychiatry*, *18*(1), 65–74. https://doi.org/10.36131/cnfioritieditore20210106

25. Gómez Galán, J., Lázaro-Pérez, C., & Martínez-López, J. Á. (2021). Exploratory study on video game addiction of college students in a pandemic scenario. *Journal of New Approaches in Educational Research*, *10*(2), 330. https://doi.org/10.7821/naer.2021.7.750

26. Hall, L. C., Drummond, A., Sauer, J. D., & Ferguson, C. J. (2021). Effects of self-isolation and quarantine on loot box spending and excessive gaming—Results of a natural experiment. *PeerJ*, *9*, e10705. https://doi.org/10.7717/peerj.10705

27. Higuchi, S., Mihara, S., Kitayuguchi, T., Miyakoshi, H., Ooi, M., Maezono, M., Nishimura, K., & Matsuzaki, T. (2020). Prolonged use of Internet and gaming among

treatment seekers arising out of social restrictions related to COVID-19 pandemic. *Psychiatry and Clinical Neurosciences*, *74*(11), 607–608. https://doi.org/10.1111/pcn.13127

28. Ismail, N., Tajjudin, A. I., Jaafar, H., Nik Jaafar, N. R., Baharudin, A., & Ibrahim, N. (2021). The relationship between Internet addiction, Internet gaming and anxiety among medical students in a Malaysian public university during COVID-19 pandemic. *International Journal of Environmental Research and Public Health*, *18*(22), 11870. https://doi.org/10.3390/ijerph182211870

29. Kim, D., & Lee, J. (2021). Addictive Internet gaming usage among Korean adolescents before and after the outbreak of the COVID-19 pandemic: A comparison of the latent profiles in 2018 and 2020. *International Journal of Environmental Research and Public Health*, *18*(14), 7275. https://doi.org/10.3390/ijerph18147275

30. Kim, D., Lee, J., & Nam, J. K. (2021). Latent profile of Internet and Internet game usage among South Korean adolescents during the COVID-19 pandemic. *Frontiers in Psychiatry*, *12*, 714301. https://doi.org/10.3389/fpsyt.2021.714301

31. Koós, M., Demetrovics, Z., Griffiths, M. D., & Bőthe, B. (2022). No significant changes in addictive and problematic behaviors during the COVID-19 pandemic and related lockdowns: A three-wave longitudinal study. *Frontiers in Psychology*, *13*, 837315. https://doi.org/10.3389/fpsyg.2022.837315

32. Maraz, A., Katzinger, E., & Yi, S. (2021). Potentially addictive behaviours increase during the first six months of the Covid-19 pandemic. *Journal of Behavioral Addictions*, *10*(4), 912–919. https://doi.org/10.1556/2006.2021.00079

33. Müller, S. M., Wegmann, E., Garcia Arías, M., Bernabéu Brotóns, E., Marchena Giráldez, C., & Brand, M. (2022). Decision making and risk propensity in individuals with tendencies towards specific Internet-use disorders. *Brain Sciences*, *12*(2), 201. https://doi.org/10.3390/brainsci12020201

34. Naaj, M. A., & Nachouki, M. (2021). Distance education during the COVID-19 pandemic: The impact of online gaming addiction on university students' performance. *International Journal of Advanced Computer Science and Applications*, *12*(9). https://doi.org/10.14569/IJACSA.2021.0120941

35. Nugraha, Y. P., Awalya, A., & Mulawarman, M. (2021a). Predicting video game addiction: The effects of composite regulatory focus and interpersonal competence among Indonesian teenagers during COVID-19 pandemic. *Islamic Guidance and Counseling Journal*, *4*(1), 66–77. https://doi.org/10.25217/igcj.v4i1.1199

36. Nugraha, Y. P., Awalya, A., & Mulawarman, M. (2021b). Video game addiction among students during COVID-19 pandemic based on regulatory focus theory and interpersonal competence. *Addictive Disorders & Their Treatment*, *20*(4), 242–249. https://doi.org/10.1097/ADT.0000000000000262

37. Oka, T., Hamamura, T., Miyake, Y., Kobayashi, N., Honjo, M., Kawato, M., Kubo, T., & Chiba, T. (2021). Prevalence and risk factors of internet gaming disorder and problematic Internet use before and during the COVID-19 pandemic: A large online survey of Japanese adults. *Journal of Psychiatric Research*, *142*, 218–225. https://doi.org/10.1016/j.jpsychires.2021.07.054

38. Oliveira, T. D. O., Costa, D. S., Alvim-Soares, A., de Paula, J. J., Kestelman, I., Silva, A. G., Malloy-Diniz, L. F., & Miranda, D. M. (2022). Children's behavioral problems, screen

time, and sleep problems' association with negative and positive parenting strategies during the COVID-19 outbreak in Brazil. *Child Abuse & Neglect*, *130*, 105345. https://doi.org/10.1016/j.chiabu.2021.105345

39. Paschke, K., Austermann, M. I., Simon-Kutscher, K., & Thomasius, R. (2021). Adolescent gaming and social media usage before and during the COVID-19 pandemic: Interim results of a longitudinal study. *SUCHT*, *67*(1), 13–22. https://doi.org/10.1024/0939-5911/a000694
40. Phuah Rong Yao, J., Sundar, V., & Ramalingam, V. (2021). Predictors of mobile video gaming on musculoskeletal pain among university students in Selangor, Malaysia. *Revista Pesquisa em Fisioterapia*, *11*(3), 518–527. https://doi.org/10.17267/2238-2704rpf.v11i3.3916
41. Rodda, S. N., Park, J. J., Wilkinson-Meyers, L., & King, D. L. (2022). The impact of COVID-19 on addiction treatment in New Zealand. *Addictive Behaviors*, *127*, 107230. https://doi.org/10.1016/j.addbeh.2021.107230
42. Rozgonjuk, D., Pontes, H. M., Schivinski, B., & Montag, C. (2022). Disordered gaming, loneliness, and family harmony in gamers before and during the COVID-19 pandemic. *Addictive Behaviors Reports*, *15*, 100426. https://doi.org/10.1016/j.abrep.2022.100426
43. Sallie, S. N., Ritou, V. J. E., Bowden-Jones, H., & Voon, V. (2021). Assessing online gaming and pornography consumption patterns during COVID-19 isolation using an online survey: Highlighting distinct avenues of problematic internet behavior. *Addictive Behaviors*, *123*, 107044. https://doi.org/10.1016/j.addbeh.2021.107044
44. Saritepeci, M., Yildiz Durak, H., & Atman Uslu, N. (2022). A latent profile analysis for the study of multiple screen addiction, mobile social gaming addiction, general mattering, and

family sense of belonging in university students. *International Journal of Mental Health and Addiction*. https://doi.org/10.1007/s11469-022-00816-y

45. Savolainen, I., Vuorinen, I., Sirola, A., & Oksanen, A. (2022). Gambling and gaming during COVID-19: The role of mental health and social motives in gambling and gaming problems. *Comprehensive Psychiatry*, *117*, 152331. https://doi.org/10.1016/j.comppsych.2022.152331
46. She, R., Wong, K., Lin, J., Leung, K., Zhang, Y., & Yang, X. (2021). How COVID-19 stress related to schooling and online learning affects adolescent depression and Internet gaming disorder: Testing Conservation of Resources theory with sex difference. *Journal of Behavioral Addictions*, *10*(4), 953–966. https://doi.org/10.1556/2006.2021.00069
47. She, R., Wong, K., Lin, J., Zhang, Y., Leung, K., & Yang, X. (2022). Profiles of stress and coping associated with mental, behavioral, and Internet use problems among adolescents During the COVID-19 pandemic: A stratified random sampling and cluster analysis. *Frontiers in Public Health*, *10*, 826911. https://doi.org/10.3389/fpubh.2022.826911
48. Shrestha, M. V., Manandhar, N., Sharma, S. C., & Joshi, S. K. (2020). Gaming disorder among medical college students during COVID-19 pandemic lockdown. *Kathmandu University Medical Journal*, *18*(2), 48–52. https://doi.org/10.3126/kumj.v18i2.32956
49. Singh, S., Datta, M., Gupta, P., & Batra, S. (2022). Predictors of 'problematic Internet use' among adolescents and adults amid the pandemic in India. *Clinical Epidemiology and Global Health*, *15*, 101036. https://doi.org/10.1016/j.cegh.2022.101036
50. Son, S., Oh, J., & Jeon, B. (2021). Correlational study between online friendship network and Internet game disorder among university students. *Brain and Behavior*, *11*(11). https://doi.org/10.1002/brb3.2392

51. Teng, Z., Pontes, H. M., Nie, Q., Griffiths, M. D., & Guo, C. (2021). Depression and anxiety symptoms associated with Internet gaming disorder before and during the COVID-19 pandemic: A longitudinal study. *Journal of Behavioral Addictions*, *10*(1), 169–180. https://doi.org/10.1556/2006.2021.00016

52. Ting, C. H., & Essau, C. (2021). Addictive behaviours among university students in Malaysia during COVID-19 pandemic. *Addictive Behaviors Reports*, *14*, 100375. https://doi.org/10.1016/j.abrep.2021.100375

53. Tzang, R.-F., Chang, C.-H., & Chang, Y.-C. (2022). Structural Equation Modeling (SEM): Gaming disorder leading untreated Attention-Deficit/Hyperactivity Disorder to disruptive mood dysregulation. *International Journal of Environmental Research and Public Health*, *19*(11), 6648. https://doi.org/10.3390/ijerph19116648

54. Volpe, U., Orsolini, L., Salvi, V., Albert, U., Carmassi, C., Carrà, G., Cirulli, F., Dell'Osso, B., Luciano, M., Menculini, G., Nanni, M., Pompili, M., Sani, G., Sampogna, G., Group, W., & Fiorillo, A. (2022). COVID-19-related social isolation predispose to problematic Internet and online video gaming use in Italy. *International Journal of Environmental Research and Public Health*, *19*(3), 1539. https://doi.org/10.3390/ijerph19031539

55. Wang, Y., Liu, B., Zhang, L., & Zhang, P. (2022). Anxiety, depression, and stress are associated with Internet gaming disorder during COVID-19: Fear of missing out as a mediator. *Frontiers in Psychiatry*, *13*, 827519. https://doi.org/10.3389/fpsyt.2022.827519

56. Werling, A. M., Walitza, S., Eliez, S., & Drechsler, R. (2022). Impact of the COVID-19 pandemic on mental health and family situation of clinically referred children and adolescents in Switzerland: Results of a survey among mental health care professionals

after 1 year of COVID-19. *Journal of Neural Transmission*, *129*(5–6), 675–688. https://doi.org/10.1007/s00702-022-02512-6

57. Wu, Q., Luo, T., Tang, J., Wang, Y., Wu, Z., Liu, Y., Chen, W., Deng, Q., & Liao, Y. (2022). Gaming in China before the COVID-19 pandemic and after the lifting of lockdowns: A nationwide online retrospective survey. *International Journal of Mental Health and Addiction*. https://doi.org/10.1007/s11469-022-00792-3

58. Xiang, G.-X., Gan, X., Jin, X., Zhang, Y.-H., & Zhu, C.-S. (2022). Developmental assets, self-control and Internet gaming disorder in adolescence: Testing a moderated mediation model in a longitudinal study. *Frontiers in Public Health*, *10*, 808264. https://doi.org/10.3389/fpubh.2022.808264

59. Yang, X., Yip, B. H. K., Lee, E. K. P., Zhang, D., & Wong, S. Y. S. (2021). The relationship between technology use and problem technology use and potential psychosocial mechanisms: Population-based telephone survey in community adults during COVID-19. *Frontiers in Psychology*, *12*, 696271. https://doi.org/10.3389/fpsyg.2021.696271

60. Zaman, M., Babar, M. S., Babar, M., Sabir, F., Ashraf, F., Tahir, M. J., Ullah, I., Griffiths, M. D., Lin, C.-Y., & Pakpour, A. H. (2022). Prevalence of gaming addiction and its impact on sleep quality: A cross-sectional study from Pakistan. *Annals of Medicine and Surgery*, *78*, 103641. https://doi.org/10.1016/j.amsu.2022.103641

61. Zarco-Alpuente, A., Ciudad-Fernández, V., Ballester-Arnal, R., Billieux, J., Gil-Llario, M. D., King, D. L., Montoya-Castilla, I., Samper, P., & Castro-Calvo, J. (2021). Problematic Internet use prior to and during the COVID-19 pandemic. *Cyberpsychology: Journal of Psychosocial Research on Cyberspace*, *15*(4). https://doi.org/10.5817/CP2021-4-1

62. Zhu, S., Zhuang, Y., Lee, P., Li, J. C.-M., & Wong, P. W. C. (2021). Leisure and problem gaming behaviors among children and adolescents during school closures caused by COVID-19 in Hong Kong: Quantitative cross-sectional survey study. *JMIR Serious Games*, *9*(2), e26808. https://doi.org/10.2196/26808
