# Supplementary material for: Problematic Gaming during COVID-19 Pandemic: A Systematic Review, Meta-Analysis, and Meta-Regression
Source: Healthcare (Basel). 2023 Dec 15;11(24):3176. doi: 10.3390/healthcare11243176 (PMC10742917; doi:10.3390/healthcare11243176)
Supplement: Supplementary file 1 [file healthcare-11-03176-s001.zip › S5 - Excluded.pdf]

Supplementary S5. List of excluded references ($N = 86$) and reason for exclusion.

1. (wrong outcome) Akozlu, Z., Kolukisa, T., Ozturk Sahin, O., & Topan, A. (2021). Internet addiction and stressors causing Internet addiction in primary school children during the COVID-19 pandemic: A descriptive and cross-sectional study from Turkey. *ADDICTA: The Turkish Journal on Addictions*, *8*(1), 65–72. https://doi.org/10.5152/ADDICTA.2021.21032
2. (wrong publication type) Albarello, F., Novoa, Á., Castro Sánchez, M., Velasco, A., Novaro Hueyo, M. V., & Narbais, F. (2021). The social dynamics of multiplayer online videogames in Argentinian and Chilean family contexts: The case of Fortnite. *Global Studies of Childhood*, *11*(4), 302–317. https://doi.org/10.1177/20436106211015997
3. (wrong publication type) Amin, K. P., Griffiths, M. D., & Dsouza, D. D. (2022). Online gaming during the COVID-19 pandemic in India: Strategies for work-life balance. *International Journal of Mental Health and Addiction*, *20*(1), 296–302. https://doi.org/10.1007/s11469-020-00358-1
4. (wrong study design) Arend, A.-K., Blechert, J., Pannicke, B., & Reichenberger, J. (2021). Increased screen use on days with increased perceived COVID-19-related confinements—A day level ecological momentary assessment study. *Frontiers in Public Health*, *8*, 623205. https://doi.org/10.3389/fpubh.2020.623205
5. (wrong outcome) Ballard, M. E., & Spencer, M. T. (2022). Importance of social videogaming for connection with others during the COVID-19 pandemic. *Games and Culture*, 155541202210909. https://doi.org/10.1177/15554120221090982

6. (wrong outcome) Barr, M., & Copeland-Stewart, A. (2022). Playing video games during the COVID-19 pandemic and effects on players' well-being. *Games and Culture*, *17*(1), 122–139. https://doi.org/10.1177/15554120211017036

7. (wrong population) Bellringer, M. E., & Garrett, N. (2021). Risk factors for increased online gambling during COVID-19 lockdowns in New Zealand: A longitudinal study. *International Journal of Environmental Research and Public Health*, *18*(24), 12946. https://doi.org/10.3390/ijerph182412946

8. (wrong publication type) Bengtsson, T. T., Bom, L. H., & Fynbo, L. (2021). Playing apart together: Young people's online gaming during the COVID-19 lockdown. *YOUNG*, *29*(4_suppl), S65–S80. https://doi.org/10.1177/11033088211032018

9. (wrong publication type) Blake, E., & Sauermilch, D. (2021). Reconsidering Internet gaming disorder during the COVID-19 pandemic. *Journal of Technology in Behavioral Science*, *6*(2), 348–351. https://doi.org/10.1007/s41347-020-00184-1

10. (wrong outcome) Boldi, A., Rapp, A., & Tirassa, M. (2022). Playing during a crisis: The impact of commercial video games on the reconfiguration of people's life during the COVID-19 pandemic. *Human–Computer Interaction*, 1–42. https://doi.org/10.1080/07370024.2022.2050725

11. (wrong outcome) Branquinho, C., Paiva, T., Guedes, F., Gaspar, T., Tomé, G., & Gaspar de Matos, M. (2022). Health risk behaviors before and during COVID-19 and gender differences. *Journal of Community Psychology*, *50*(2), 1102–1110. https://doi.org/10.1002/jcop.22705

12. (wrong outcome) Canale, N., Marino, C., Lenzi, M., Vieno, A., Griffiths, M. D., Gaboardi, M., Giraldo, M., Cervone, C., & Santinello, M. (2022). How communication technology

fosters individual and social wellbeing during the COVID-19 pandemic: Preliminary support for a digital interaction model. *Journal of Happiness Studies*, *23*(2), 727–745. https://doi.org/10.1007/s10902-021-00421-1

13. (wrong outcome) Chae, S. W., & Lee, S. H. (2022). Sharing emotion while spectating video game play: Exploring Twitch users' emotional change after the outbreak of the COVID-19 pandemic. *Computers in Human Behavior*, *131*, 107211. https://doi.org/10.1016/j.chb.2022.107211
14. (wrong outcome) Chockalingam, S., Gayathri, R., Yuvaraj Babu, K., & Vishnu Priya, V. (2020). Online gaming and urban school children during lockdown-a survey. *International Journal of Pharmaceutical Research*, *12*, 1613–1621. https://doi.org/10.31838/ijpr/2020.SP2.188
15. (wrong publication type) Cowan, K., Potter, J., Olusoga, Y., Bannister, C., Bishop, J. C., Cannon, M., & Signorelli, V. (2021). Children's digital play during the COVID-19 pandemic: Insights from the play observatory. *Journal of E-Learning and Knowledge Society*, *17*(3), 8–17. https://doi.org/10.31838/ijpr/2020.SP2.188
16. (wrong publication type) Coward-Gibbs, M. (2021). Why don't we play pandemic? Analog gaming communities in lockdown. *Leisure Sciences*, *43*(1), 78–84. https://doi.org/10.1080/01490400.2020.1773986
17. (wrong outcome) Coyne, P., Staffell, Z., & Woodruff, S. J. (2021). Recreational screen time use among a small sample of Canadians during the first six months of the COVID-19 pandemic. *International Journal of Environmental Research and Public Health*, *18*(23). https://doi.org/10.3390/ijerph182312664

18. (wrong outcome) Croissant, M., & Frister, M. (2021). A data-driven approach for examining the demand for relaxation games on Steam during the COVID-19 pandemic. *PLoS ONE*, *16*(12), e0261328–e0261328. https://doi.org/10.1371/journal.pone.0261328

19. (not written in English) De Rostolan, T., & Bonnaire, C. (2022). Impact of the COVID-19 pandemic on gaming use according to personality and type of passion in adults. *Psychologie Française*, S0033298422000280. https://doi.org/10.1016/j.psfr.2022.04.002

20. (wrong publication type) Emmerich, K., Krekhov, A., & Krüger, J. (2020). *"pls Uninstall": On the interplay of the COVID-19 pandemic and toxic player behavior in competitive gaming*. 224–228. https://doi.org/10.1145/3383668.3419896

21. (wrong publication type) Etchells, P. J., Davidson, B. I., Kaye, L. K., Ellis, D. A., & Lieberoth, A. (2022). Researchers should avoid causally attributing suicide to video game play as a single factor. *Perspectives in Psychiatric Care*, *58*(2), 880–882. https://doi.org/10.1111/ppc.12762

22. (wrong publication type) Fazeli, S., Zeidi, I. M., Lin, C.-Y., Namdar, P., Griffiths, M. D., Ahorsu, D. K., & Pakpour, A. (2020). Corrigendum to "Depression, anxiety, and stress mediate the associations between internet gaming disorder, insomnia, and quality of life during the COVID-19 outbreak" [Addict. Behav. Rep. 12 (2020) 100307]. *Addictive Behaviors Reports*, *12*, 100322. https://doi.org/10.1016/j.abrep.2020.100322

23. (wrong outcome) Fernandes, B., Uzun, B., Aydin, C., Tan-Mansukhani, R., Vallejo, A., Saldaña-Gutierrez, A., Nanda Biswas, U., & Essau, C. A. (2021). Internet use during COVID-19 lockdown among young people in low- and middle-income countries: Role of psychological wellbeing. *Addictive Behaviors Reports*, *14*, 100379–100379. https://doi.org/10.1016/j.abrep.2021.100379

24. (wrong outcome) Fontana, M. T. (2020). Gamification of ChemDraw during the COVID-19 pandemic: Investigating how a serious, educational-game tournament (Molecule Madness) impacts student wellness and organic chemistry skills while distance learning. *Journal of Chemical Education*, *97*(9), 3358–3368. https://doi.org/10.1021/acs.jchemed.0c00722
25. (wrong outcome) Formosa, J., Johnson, D., Türkay, S., & Mandryk, R. L. (2022). Need satisfaction, passion and wellbeing effects of videogame play prior to and during the COVID-19 pandemic. *Computers in Human Behavior*, *131*, 107232–107232. https://doi.org/10.1016/j.chb.2022.107232
26. (wrong outcome) Fraser, A. M., Stockdale, L. A., Bryce, C. I., & Alexander, B. L. (2022). College students' media habits, concern for themselves and others, and mental health in the era of COVID-19. *Psychology of Popular Media*, *11*(2), 139–151. https://doi.org/10.1037/ppm0000345
27. (wrong outcome) Freeland, L., O'reilly, M., Fleury, J., Adams, S., & Vostanis, P. (2022). Digital social and emotional literacy intervention for vulnerable children in Brazil: Participants' experiences. *International Journal of Mental Health Promotion*, *24*(1), 51–67. https://doi.org/10.32604/ijmhp.2022.015706
28. (wrong publication type) Gega, L., & Aboujaoude, E. (2021). How digital technology mediated the effects of the COVID-19 pandemic on mental health: The good, the bad, and the indifferent. *Frontiers in Digital Health*, *3*, 733151–733151. https://doi.org/10.3389/fdgth.2021.733151
29. (wrong outcome) Gómez-Baya, D., Grasmeijer, A. J., López-Bermúdez, E., Gaspar de Matos, M., & Mendoza, R. (2022). Positive youth development and Internet use in a

sample of Spanish adolescents. *Frontiers in Pediatrics*, *10*, 842928–842928. https://doi.org/10.3389/fped.2022.842928

30. (wrong publication type) Handique, K., & Teronpi, J. (2022). *A study on Internet addiction disorder (IAD) and its related psychological factors during the COVID-19 lockdown* (rayyan-840249103). *107*(1), 16323–16341. https://doi.org/10.1149/10701.16323ecst
31. (wrong outcome) Haug, E., Mæland, S., Lehmann, S., Bjørknes, R., Fadnes, L. T., Sandal, G. M., & Skogen, J. C. (2022). Increased gaming during COVID-19 predicts physical inactivity among youth in Norway—A two-wave longitudinal cohort study. *Frontiers in Public Health*, *10*, 812932–812932. https://doi.org/10.3389/fpubh.2022.812932
32. (wrong outcome) Helbach, J., & Stahlmann, K. (2021). Changes in digital media use and physical activity in german young adults under the covid-19 pandemic: A cross-sectional study. *Journal of Sports Science and Medicine*, *20*(4), 642–654. https://doi.org/10.52082/jssm.2021.642
33. (wrong study design) Hunt, D. (2022). Microtransaction spending and problematic gambling of UK university Call of Duty gamers. *International Gambling Studies*. https://doi.org/10.1080/14459795.2022.2046843
34. (wrong outcome) Kattula, D., Singh Balhara, Y. P., Chukkali, S., Singh, S., Bhargava, R., & Ganesh, R. (2021). Screen time beyond gaming and social media: Excessive and problematic use of Over The Top (OTT) platforms among college students during COVID-19 pandemic. *Psychiatria Danubina*, *33*(Suppl 13), 420–423.
35. (wrong outcome) Kim, M. (2021). Does playing a video game really result in improvements in psychological well-being in the era of COVID-19? *Journal of Retailing and Consumer Services*, *61*, 102577. https://doi.org/10.1016/j.jretconser.2021.102577

36. (wrong publication type) Kim, Y. H., Nauright, J., & Suveatwatanakul, C. (2020). The rise of E-Sports and potential for Post-COVID continued growth. *Sport in Society*, *23*(11), 1861–1871. https://doi.org/10.1080/17430437.2020.1819695

37. (wrong publication type) King, D. L., Achab, S., Higuchi, S., Bowden-Jones, H., Müller, K. W., Billieux, J., Starcevic, V., Saunders, J. B., Tam, P., & Delfabbro, P. H. (2022). Gaming disorder and the COVID-19 pandemic: Treatment demand and service delivery challenges. *Journal of Behavioral Addictions*, *11*(2), 243–248. https://doi.org/10.1556/2006.2022.00011

38. (wrong publication type) King, D. L., Delfabbro, P. H., Billieux, J., & Potenza, M. N. (2020). Problematic online gaming and the COVID-19 pandemic. *Journal of Behavioral Addictions*, *9*(2), 184–186. https://doi.org/10.1556/2006.2020.00016

39. (wrong publication type) Király, O., Potenza, M. N., Stein, D. J., King, D. L., Hodgins, D. C., Saunders, J. B., Griffiths, M. D., Gjoneska, B., Billieux, J., Brand, M., Abbott, M. W., Chamberlain, S. R., Corazza, O., Burkauskas, J., Sales, C. M. D., Montag, C., Lochner, C., Grünblatt, E., Wegmann, E., … Demetrovics, Z. (2020). Preventing problematic Internet use during the COVID-19 pandemic: Consensus guidance. *Comprehensive Psychiatry*, *100*, 152180. https://doi.org/10.1016/j.comppsych.2020.152180

40. (wrong publication type) Ko, C.-H., & Yen, J.-Y. (2020). Impact of COVID-19 on gaming disorder: Monitoring and prevention. *Journal of Behavioral Addictions*, *9*(2), 187–189. https://doi.org/10.1556/2006.2020.00040

41. (wrong publication type) Kowal, M., Conroy, E., Ramsbottom, N., Smithies, T., Toth, A., & Campbell, M. (2021). Gaming your mental health: A narrative review on mitigating

symptoms of depression and anxiety using commercial video games. *JMIR Serious Games*, *9*(2), e26575. https://doi.org/10.2196/26575

42. (wrong outcome) Krittanawong, C., Virk, H. U. H., Katz, C. L., Kaplin, S., Wang, Z., Gonzalez-Heydrich, J., Storch, E. A., & Lavie, C. J. (2022). Association of social gaming with well-being (Escape COVID-19): A sentiment analysis. *The American Journal of Medicine*, *135*(2), 254–257. https://doi.org/10.1016/j.amjmed.2021.10.010
43. (wrong publication type) Lamar, L., Craig, J. T., & Shubkin, C. D. (2021). So your patient is a gamer: An exploration of benefits and harms. *Current Opinion in Pediatrics*, *33*(5), 530–534. https://doi.org/10.1097/MOP.0000000000001053
44. (wrong outcome) Laniton, J. M., Vallar, J. B., & Cuarteros, K. G. (2022). University students' viewpoints: A coping mechanism amid the Covid-19 pandemic. *Canadian Journal of Family and Youth / Le Journal Canadien de Famille et de La Jeunesse*, *14*(3), 20–31. https://doi.org/10.29173/cjfy29790
45. (wrong outcome) Lemenager, T., Neissner, M., Koopmann, A., Reinhard, I., Georgiadou, E., Müller, A., Kiefer, F., & Hillemacher, T. (2020). COVID-19 lockdown restrictions and online media consumption in Germany. *International Journal of Environmental Research and Public Health*, *18*(1), 14. https://doi.org/10.3390/ijerph18010014
46. (not written in English) Lesimple, P., Verley, J.-B., Laplace, B., & Olliac, B. (2022). Jeux vidéo à l'adolescence: Paradoxes en temps de pandémie. *Neuropsychiatrie de l'Enfance et de l'Adolescence*, *70*(1), 37–42. https://doi.org/10.1016/j.neurenf.2021.10.002
47. (wrong outcome) Li, X., Vanderloo, L. M., Keown-Stoneman, C. D. G., Cost, K. T., Charach, A., Maguire, J. L., Monga, S., Crosbie, J., Burton, C., Anagnostou, E., Georgiades, S., Nicolson, R., Kelley, E., Ayub, M., Korczak, D. J., & Birken, C. S. (2021).

Screen use and mental health symptoms in Canadian children and youth during the COVID-19 pandemic. *JAMA Network Open*, *4*(12), e2140875. https://doi.org/10.1001/jamanetworkopen.2021.40875

48. (wrong publication type) Limone, P., & Toto, G. A. (2021). Psychological and emotional effects of digital technology on children in COVID-19 pandemic. *Brain Sciences*, *11*(9), 1126. https://doi.org/10.3390/brainsci11091126

49. (wrong outcome) López-Cabarcos, M. Á., Ribeiro-Soriano, D., & Piñeiro-Chousa, J. (2020). All that glitters is not gold. The rise of gaming in the COVID-19 pandemic. *Journal of Innovation & Knowledge*, *5*(4), 289–296. https://doi.org/10.1016/j.jik.2020.10.004

50. (wrong publication type) Mahapatra, S., Sharma, M., Amudhan, S., & Anand, N. (2021). Binge gaming and COVID-19: A looming crisis. *Journal of Mental Health and Human Behaviour*, *26*(1), 83–83. https://doi.org/10.4103/jmhhb.jmhhb_26_21

51. (wrong population) Maldonado-Murciano, L., Guilera, G., Montag, C., & Pontes, H. M. (2022). Disordered gaming in esports: Comparing professional and non-professional gamers. *Addictive Behaviors*, *132*, 107342. https://doi.org/10.1016/j.addbeh.2022.107342

52. (wrong study design) Mamun, M. A., Ullah, I., Usman, N., & Griffiths, M. D. (2022). PUBG-related suicides during the COVID-19 pandemic: Three cases from Pakistan. *Perspectives in Psychiatric Care*, *58*(2), 877–879. https://doi.org/10.1111/ppc.12640

53. (wrong outcome) Manzano-León, A., Rodríguez-Ferrer, J. M., Aguilar-Parra, J. M., & Herranz-Hernández, R. (2022). Gamification and family leisure to alleviate the psychological impact of confinement due to COVID-19. *Children & Society*, *36*(4), 433–449. https://doi.org/10.1111/chso.12495

54. (wrong outcome) Meriläinen, M. (2022). Pandemic rhythms: Adults' gaming in Finland during the spring 2020 COVID-19 restrictions. *Convergence*. https://doi.org/10.1177/13548565221077582

55. (wrong outcome) Ming, Y., Hongxian, S., & Wei, H. (2021). COVID-19 and the risk of gaming disorder: A reflection in the situation of regular epidemic prevention and control. *Chinese Journal of Psychiatry*, *54*(2), 81–86.

56. (wrong outcome) Mingazova, E. N., Akimova, L. V., Moscow Center «Patriot. Sport», 107370, Moscow, Russia, Pozharskaya, E. N., Don State Technical University, 344000, Rostov-on-Don, Russia, Mingazov, R. N., & A. N. Kosygin Russian State University, 117997, Moscow, Russia. (2021). Health risks of school-age children with distance learning in the first months of the spread of COVID-19. *Problems of Social Hygiene Public Health and History of Medicine*, *29*(Special Issue). https://doi.org/10.32687/0869-866X-2021-29-s1-588-592

57. (wrong outcome) Minian, N., Saiva, A., Gayapersad, A., Dragonetti, R., Proulx, C., Debergue, P., Lecce, J., Hussain, S., Desjardins, E., & Selby, P. (2022). Video game to attenuate pandemic-related stress from an equity lens: Development and usability study. *JMIR Formative Research*, *6*(5), e36820–e36820. https://doi.org/10.2196/36820

58. (wrong outcome) Nan, D., Lee, H., Kim, Y., & Kim, J. H. (2022). My video game console is so cool! A coolness theory-based model for intention to use video game consoles. *Technological Forecasting and Social Change*, *176*, 121451. https://doi.org/10.1016/j.techfore.2021.121451

59. (wrong publication type) Navarro, J. (2021). Fortnite: A context for child development in the U.S. during COVID-19 (and beyond). *Journal of Children and Media*, *15*(1), 13–16. https://doi.org/10.1080/17482798.2020.1858435

60. (wrong outcome) Nilsson, A., Rosendahl, I., & Jayaram-Lindström, N. (2022). Gaming and social media use among adolescents in the midst of the COVID-19 pandemic. *Nordic Studies on Alcohol and Drugs*, 145507252210749. https://doi.org/10.1177/14550725221074997

61. (wrong outcome) Obana, T., Takubo, M., Orito, Y., Murata, K., Sai, H., & Okamoto, T. (2021). The online attention game for digital identity education: An exploratory study. *The Review of Socionetwork Strategies*, *15*(1), 251–276. https://doi.org/10.1007/s12626-021-00077-6

62. (wrong outcome) Oflu, A., Bükülmez, A., Elmas, E., Tahta, E. G., & Çeleğen, M. (2021). Comparison of screen time and digital gaming habits of Turkish children before and during the coronavirus disease 2019 pandemic. *Turkish Archives of Pediatrics*, *56*(1), 22–26. https://doi.org/10.14744/TurkPediatriArs.2020.41017

63. (wrong publication type) O'Loughlin, J., Pelletier, E., Loomis, E., & Alweis, R. (2022). A case of Internet gaming disorder: When #PlayApartTogether takes a dark turn. *Journal of Community Hospital Internal Medicine Perspectives*, *12*(1), 32–35. https://doi.org/10.55729/2000-9666.1005

64. (wrong publication type) Pallavicini, F., Pepe, A., & Mantovani, F. (2022). The effects of playing video games on stress, anxiety, depression, loneliness, and gaming disorder during the early stages of the COVID-19 pandemic: PRISMA systematic review.

Cyberpsychology, Behavior, and Social Networking, *25*(6), 334–354. https://doi.org/10.1089/cyber.2021.0252

65. (not written in English) Pérez-Albéniz, A., Nuez, C., Lucas-Molina, B., Ezquerra, M., & Fonseca-Pedrero, E. (2022). Impact of lockdown on the addictive behavior of university students in La Rioja. *Adicciones*. https://doi.org/10.20882/adicciones.1646
66. (wrong outcome) Pop-Jordanova, N., & Loleska, S. (2021). Neuropsychological correlates of Internet addiction. *Prilozi*, *42*(3), 17–28. https://doi.org/10.2478/prilozi-2021-0031
67. (wrong outcome) Prochnow, T., Hartnell, L., & Patterson, M. S. (2021). Depressive symptoms, developing friendships, and social support through online gaming: A mixed-method analysis of online gaming network communication. *Mental Health and Social Inclusion*, *25*(3), 243–253. https://doi.org/10.1108/MHSI-02-2021-0011
68. (wrong publication type) Rahmawati, N. A., Setiawati, Y., Indah Ardani, G. A., Zain, E., & Pereira-Sanchez, V. (2022). Internet gaming disorder in an adolescent during the COVID-19 pandemic: A case report. *Pan African Medical Journal*, *41*. https://doi.org/10.11604/pamj.2022.41.224.33941
69. (wrong outcome) Reuter, P. R., Forster, B. L., & Kruger, B. J. (2021). A longitudinal study of the impact of COVID-19 restrictions on students' health behavior, mental health and emotional well-being. *PeerJ*, *9*, e12528. https://doi.org/10.7717/peerj.12528
70. (wrong publication type) Rogier, G., Zobel, S. B., & Velotti, P. (2021). COVID-19 and technological addiction: The role of loneliness. *European Psychiatry*, *64*(S1), S560–S560. https://doi.org/10.1192/j.eurpsy.2021.1493
71. (wrong outcome) Ruiz-Palmero, J., Colomo-Magaña, E., Sánchez-Rivas, E., & Linde-Valenzuela, T. (2021). Study of the use and consumption of mobile devices in university

students. *Digital Education Review*, *39*, 89–104. https://doi.org/10.1344/der.2021.39.89-104

72. (wrong publication type) Rumpf, H.-J., Brand, M., Wegmann, E., Montag, C., Müller, A., Müller, K., Wölfling, K., Stark, R., Steins-Löber, S., Hayer, T., Schlossarek, S., Hoffmann, H., Leménager, T., Lindenberg, K., Thomasius, R., Batra, A., Mann, K., te Wildt, B., Mößle, T., & Rehbein, F. (2020). The COVID-19 pandemic and behavioral addiction-New challenges for structural and behavioral prevention. *SUCHT*, *66*(4), 212–216. https://doi.org/10.1024/0939-5911/a000672
73. (wrong outcome) Sciberras, E., Patel, P., Stokes, M. A., Coghill, D., Middeldorp, C. M., Bellgrove, M. A., Becker, S. P., Efron, D., Stringaris, A., Faraone, S. V., Bellows, S. T., Quach, J., Banaschewski, T., McGillivray, J., Hutchinson, D., Silk, T. J., Melvin, G., Wood, A. G., Jackson, A., … Westrupp, E. (2022). Physical health, media use, and mental health in children and adolescents with ADHD during the COVID-19 pandemic in Australia. *Journal of Attention Disorders*, *26*(4), 549–562. https://doi.org/10.1177/1087054720978549
74. (wrong outcome) Seguin, D., Kuenzel, E., Morton, J. B., & Duerden, E. G. (2021). School's out: Parenting stress and screen time use in school-age children during the COVID-19 pandemic. *Journal of Affective Disorders Reports*, *6*, 100217–100217. https://doi.org/10.1016/j.jadr.2021.100217
75. (wrong publication type) Shahid, R., Kumari, S., & Doumas, S. (2021). COVID-19's impact on Internet gaming disorder among children and adolescents. *Current Psychiatry*, *20*(7), 41–42. https://doi.org/10.12788/cp.0152

76. (wrong publication type) Shumaker, D., & Manning, C. (2021). Existential implications of internet gaming disorder (IGD). *The Humanistic Psychologist*. https://doi.org/10.1037/hum0000265

77. (wrong publication type) Situmorang, D. D. B. (2021). Expressive art therapies for treating online games addiction in COVID-19 outbreak: Why are they important? *Ethics, Medicine and Public Health*, *18*, 100691. https://doi.org/10.1016/j.jemep.2021.100691

78. (wrong outcome) Ueno, C., & Yamamoto, S. (2022). The relationship between behavioral problems and screen time in children during COVID-19 school closures in Japan. *Scandinavian Journal of Child and Adolescent Psychiatry and Psychology*, *10*(1), 1–8. https://doi.org/10.21307/sjcapp-2022-001

79. (wrong outcome) Vidal, I. M. G. (2021). Prevalence of the student's gender in their daily interactions with devices on the Internet. *Revista Española de Educación Comparada*, *39*, 254–270. https://doi.org/10.5944/reec.39.2021.27577

80. (wrong outcome) Vuong, Q.-H., Ho, M.-T., La, V.-P., Le, T.-T., Nguyen, T. H. T., & Nguyen, M.-H. (2021). A multinational data set of game players' behaviors in a virtual world and environmental perceptions. *Data Intelligence*, *3*(4), 606–630. https://doi.org/10.1162/dint_a_00111

81. (wrong outcome) Werling, A. M., Walitza, S., & Drechsler, R. (2021). Impact of the COVID-19 lockdown on screen media use in patients referred for ADHD to child and adolescent psychiatry: An introduction to problematic use of the internet in ADHD and results of a survey. *Journal of Neural Transmission*, *128*(7), 1033–1043. https://doi.org/10.1007/s00702-021-02332-0

82. (wrong outcome) Werling, A. M., Walitza, S., Grünblatt, E., & Drechsler, R. (2021). Media use before, during and after COVID-19 lockdown according to parents in a clinically referred sample in child and adolescent psychiatry: Results of an online survey in Switzerland. *Comprehensive Psychiatry*, *109*, 152260. https://doi.org/10.1016/j.comppsych.2021.152260

83. (wrong outcome) Werner, A. M., Petersen, J., Müller, K. W., Tibubos, A. N., Schäfer, M., Mülder, L. M., Reichel, J. L., Heller, S., Dietz, P., Wölfling, K., & Beutel, M. E. (2021). Prevalence of Internet addiction before and during the COVID-19 pandemic among students at Johannes Gutenberg University Mainz, Germany. *Suchttherapie*, *22*(04), 183–193. https://doi.org/10.1055/a-1653-8186

84. (wrong outcome) Wu, Q., Chen, T., Zhong, N., Bao, J., Zhao, Y., Du, J., & Zhao, M. (2022). Changes of Internet behavior of adolescents across the period of COVID-19 pandemic in China. *Psychology, Health & Medicine*, 1–11. https://doi.org/10.1080/13548506.2021.2019809

85. (wrong outcome) Yee, A. Z. H., & Sng, J. R. H. (2022). Animal crossing and COVID-19: A qualitative study examining how video games satisfy basic psychological needs during the pandemic. *Frontiers in Psychology*, *13*, 800683. https://doi.org/10.3389/fpsyg.2022.800683

86. (wrong publication type) Yousafzai, A. W., & Sherin, A. (2020). Internet gaming among children in the time of COVID-19: An emerging psychological problem. *Khyber Medical University Journal*, *12*(4), 261–262. https://doi.org/10.35845/kmuj.2020.21046

Update (5th December, 2022). *N* = 6

1. (wrong outcome) Bersani, F. S., Barchielli, B., Ferracuti, S., Panno, A., Carbone, G. A., Massullo, C., Farina, B., Corazza, O., Prevete, E., Tarsitani, L., Pasquini, M., Biondi, M., & Imperatori, C. (2022). The association of problematic use of social media and online videogames with aggression is mediated by insomnia severity: A cross-sectional study in a sample of 18- to 24-year-old individuals. *Aggressive Behavior*, *48*(3), 348–355. https://doi.org/10.1002/ab.22008
2. (wrong outcome) Chen, I., Chen, H.-P., Gamble, J. H., Liao, X. L., Chen, X.-M., Yang, Y.-T. C., Pakpour, A. H., Griffiths, M. D., & Lin, C.-Y. (2022). Evaluating a cross-lagged panel model between problematic internet use and psychological distress and cross-level mediation of school administrator support on problematic internet use: The serial mediating role of psychological needs thwarting of online teaching and psychological distress. *Frontiers in Public Health*, *10*, 987366. Scopus. https://doi.org/10.3389/fpubh.2022.987366
3. (wrong outcome) Jouhki, H., Savolainen, I., Sirola, A., & Oksanen, A. (2022). Escapism and excessive online behaviors: A three-wave longitudinal study in Finland during the COVID-19 pandemic. *International Journal of Environmental Research and Public Health*, *19*(19). https://doi.org/10.3390/ijerph191912491
4. (wrong outcome) Sousa Tavares, H. L., Souza Costa, D., Soares, A. A., Kestelman, I., da Silva, A. G., Malloy-Diniz, L. F., de Paula, J. J., & de Miranda, D. M. (2022). Gaming addiction and screen time in a context of increase of internalizing symptoms: Moderation evaluation. *Clinical Child Psychology and Psychiatry*, 13591045221125328. Scopus. https://doi.org/10.1177/13591045221125329

5. (wrong outcome) Xiang, G., Gan, X., Jin, X., & Zhang, Y. (2022). The more developmental assets, the less internet gaming disorder? Testing the cumulative effect and longitudinal mechanism during the COVID-19 pandemic. *Current Psychology*, 1–12. https://doi.org/10.1007/s12144-022-03790-9
6. (wrong outcome) Yang, H., Huang, X., Hu, J., Thompson, J., & Flower, R. (2022). Achievements, challenges and global implications of China's carbon neutral pledge. *Frontiers of Environmental Science & Engineering*, *16*(8). https://doi.org/10.1007/s11783-022-1532-9
