# Supplementary material for: Problematic Gaming during COVID-19 Pandemic: A Systematic Review, Meta-Analysis, and Meta-Regression
Source: Healthcare (Basel). 2023 Dec 15;11(24):3176. doi: 10.3390/healthcare11243176 (PMC10742917; doi:10.3390/healthcare11243176)
Supplement: Supplementary file 1 [file healthcare-11-03176-s001.zip › S6 - Risk of Bias.pdf]

Supplementary S6 – Risk of Bias (RoB) assessment with the Newcastle-Ottawa Scale

Record	*RoB*	*Total*	*1*	*2*	*3*	*4*	*5*	*6*	*7*
(Alqassim et al., 2022)	low	6	1	1	0.5	1.5	0.5	1	0.5
(Al-Sharqi & Hasan, 2022)	high	4	1	0	0	1	0	1	1
(Balhara et al., 2020)	high	3	1	0	0	1	0	1	0
(Brailovskaia et al., 2022)	low	7	1	1	1	1	1	1	1
(C.-Y. Chen et al., 2022)	low	6	1	0.5	0.5	1.5	1	1	0.5
(C.-Y. Chen, Chen, O'Brien, et al., 2021)	low	5	1	0.5	0	1	1	1	0.5
(C.-Y. Chen, Chen, Pakpour, et al., 2021)	low	5.5	1	0.5	0	1.5	1	1	0.5
(Cakiroglu et al., 2021)	high	4	1	0	0	0.5	0.5	1	1
(Cena et al., 2022)	high	4	1	0	0	1	0	1	1
(Chang et al., 2022)	low	5	1	0	0.5	1	0.5	1	1
(Ciccarelli et al., 2022)	low	5	1	0.5	0	1	1	1	0.5
(Claesdotter-Knutsson et al., 2022)	low	6	1	0.5	0.5	1	1	1	1
(Cuong et al., 2021)	low	8	1	1	1	2	1	1	1
(De Pasquale et al., 2021)	low	5	1	0.5	0	1.5	0.5	1	0.5
(Donati et al., 2021)	low	5.5	1	0	0	2	0.5	1	1
(Efrati & Spada, 2022)	low	5	1	0	0	1.5	0.5	1	1
(Elhai et al., 2021)	low	5	1	0	0.5	1.5	0	1	1
(Elsayed, 2021)	low	5	1	0.5	0	1	0.5	1	1
(Fernandes et al., 2020)	low	5	1	0	0	1	1	1	1
(Giardina et al., 2021)	low	6	1	0.5	0	1.5	1	1	1
(Gómez Galán et al., 2021)	low	5	1	0.5	0	1.5	0	1	1
(Hall et al., 2021)	high	4.5	1	0.5	0	1	0.5	1	0.5
(Higuchi et al., 2020)	high	2	1	0	0	0	0.5	0	0.5
(I. H. Chen et al., 2021a)	low	6	1	0.5	0	1.5	1	1	1
(I. H. Chen et al., 2022a)	low	5	1	0	0.5	1.5	0.5	1	0.5
(I.-H. Chen et al., 2021b)	low	6	1	0.5	0.5	1.5	1	1	0.5
(I.-H. Chen et al., 2022b)	low	5	1	0	0.5	1	1	1	0.5
(Ismail et al., 2021)	low	5.5	1	0	0	1.5	1	1	1
(Kim & Lee, 2021)	low	5	1	0	0	1	1	1	1
(Kim et al., 2021)	low	6	1	0.5	1	1	1	1	0.5
(Koós et al., 2022)	low	5	1	0.5	0	1	0.5	1	1
(Maraz et al., 2021)	low	5	1	0	0.5	1	0.5	1	1

(Müller et al., 2022)	low	5	1	0.5	0	1	0.5	1	1
(Naaj & Nachouki, 2021)	high	2	0.5	0	0	0	0.5	1	0
(Nugraha et al., 2021a)	high	4	1	0	0	1	0.5	1	0.5
(Nugraha et al., 2021b)	high	4	1	0	0	1	0.5	1	0.5
(Oka et al., 2021)	low	5	1	0	0.5	1	0.5	1	1
(Oliveira et al., 2022)	low	6	1	0.5	0	1.5	1	1	1
(Paschke et al., 2021)	low	5	1	0	0.5	1.5	0	1	1
(Rodda et al., 2022)	high	3	0.5	0	0	0	0.5	1	1
(Rozgonjuk et al., 2022)	low	6	1	0.5	0	2	1	1	0.5
(Sallie et al., 2021)	high	4	1	0	0	1.5	0.5	1	0
(Saritepeci et al., 2022)	low	5	1	0	0	2	0	1	1
(Savolainen et al., 2022)	low	5	1	0.5	0	0.5	1	1	1
(She et al., 2021)	low	6	1	0.5	0	1.5	1	1	1
(She et al., 2022)	low	6	1	0	0	2	1	1	1
(Shrestha et al., 2020)	low	6	1	0.5	0.5	1.5	0.5	1	1
(Singh et al., 2022)	low	6	1	0.5	0.5	2	0.5	1	0.5
(Son et al., 2021)	low	5	1	0.5	0	2	0.5	1	0
(Teng et al., 2021)	low	5	1	0	0	1	1	1	1
(Ting & Essau, 2021)	low	5	1	0	0	1.5	0.5	1	1
(Tzang et al., 2022)	low	5	1	0	0	1.5	0.5	1	1
(Volpe et al., 2022)	low	5	1	0	0	1.5	0.5	1	1
(Wang et al., 2022)	low	6	1	0	0	2	1	1	1
(Werling et al., 2022)	high	3	1	0	0	0	0	1	1
(Wu et al., 2022)	low	5	0.5	0.5	0	1.5	0.5	1	1
(Xiang et al., 2022)	low	6	1	0.5	0	2	1	1	0.5
(Yang et al., 2021)	low	6	1	0.5	0	1.5	1	1	1
(Yao et al., 2021)	low	5	1	0	0	1.5	0.5	1	1
(Zaman et al., 2022)	low	7	1	1	0.5	1.5	1	1	1
(Zarco-Alpuente et al., 2021)	low	5.5	1	0	0	2	0.5	1	1
(Zhu et al., 2021)	low	7	1	1	1	1	1	1	1

Note. Level of accordance between the two reviewers = 91.32%. The reported values are the mean of the scores assigned by the two reviewers.
