# Supplementary material for: Problematic Gaming during COVID-19 Pandemic: A Systematic Review, Meta-Analysis, and Meta-Regression
Source: Healthcare (Basel). 2023 Dec 15;11(24):3176. doi: 10.3390/healthcare11243176 (PMC10742917; doi:10.3390/healthcare11243176)
Supplement: Supplementary file 1 [file healthcare-11-03176-s001.zip › S7 - Events per study.pdf]

Supplementary S7 – Events per studies

Record	*N*	*event*
(Alqassim et al., 2022)	423	17
(Balhara et al., 2020)	128	19
(Brailovskaia et al., 2022)	140	2
(Cakiroglu et al., 2021)	410	22
(Cena et al., 2022)	502	22
(Chang et al., 2022)	1305	265
(C.-Y. Chen, Chen, O'Brien, et al., 2021)	1357	14
(I. H. Chen et al., 2021)	504	3
(I.-H. Chen et al., 2021)	535	4
(C.-Y. Chen, Chen, Pakpour, et al., 2021)	2026	17
(I. Chen et al., 2022)	980	16
(C.-Y. Chen et al., 2022)	575	5
(I.-H. Chen et al., 2022)	272	3
(Ciccarelli et al., 2022)	466	1
(Claesdotter-Knutsson et al., 2022)	932	60
(Cuong et al., 2021)	2084	242
(De Pasquale et al., 2021)	162	0
(Efrati & Spada, 2022)	1813	550
(Gómez Galán et al., 2021)	310	51
(Hall et al., 2021)	1144	36
(Kim et al., 2021)	2984	114
(Koós et al., 2022)	1043	11
(Müller et al., 2022)	174	13
(Nugraha et al., 2021a)	1046	136
(Nugraha et al., 2021b)	1046	120
(Oka et al., 2021)	51246	2088
(Rozgonjuk et al., 2022)	299	41
(Sallie et al., 2021)	1344	89
(Savolainen et al., 2022)	1530	15
(She et al., 2021)	3136	464
(Shrestha et al., 2020)	260	22
(Teng et al., 2021)	1778	89

(Ting & Essau, 2021)	178	8
(Volpe et al., 2022)	1385	29
(Wu et al., 2022)	5268	264
(Yang et al., 2021)	177	15
(Zaman et al., 2022)	618	44
(Zhu et al., 2021)	2848	152
